# Preference towards HIV Self-Testing above Other Testing Options in a Sample of Men Who Have Sex with Men from Five European Countries

**DOI:** 10.3390/ijerph18094804

**Published:** 2021-04-30

**Authors:** Juan Hoyos, Tomás Maté, Juan-Miguel Guerras, Marta Donat, Cristina Agustí, Matthias Kuske, Ricardo Fuertes, Sophocles Chanos, Francois Pichon, Luis Sordo, José Pulido, María-José Belza, on behalf of the EURO HIV EDAT Group

**Affiliations:** 1 Departamento de Salud Pública y Materno-Infantil, Universidad Complutense de Madrid, 28040 Madrid, Spain; hoyosmiller@hotmail.com (J.H.); lsordo@ucm.es (L.S.); josepuli@ucm.es (J.P.); 2 Gerencia de Atención Primaria de Valladolid Este, Gerencia Regional de Salud de Castilla y León (SACYL), 47010 Valladolid, Spain; 3 CIBER Epidemiología y Salud Pública (CIBERESP), 28029 Madrid, Spain; jguerras@isciii.es (J.-M.G.); cagusti@iconcologia.net (C.A.); mbelza@isciii.es (M.-J.B.); 4 Centro Nacional de Epidemiología, Instituto de Salud Carlos III, 28029 Madrid, Spain; 5 Escuela Nacional de Sanidad, Instituto de Salud Carlos III, 28029 Madrid, Spain; ma.donat@isciii.es; 6 Departament de Salut, Centre Estudis Epidemiologics sobre les Infeccions de Transmissio Sexual i Sida de Catalunya (CEEISCAT), Generalitat de Catalunya, 08005 Badalona, Spain; 7 AIDS Hilfe NRW e.V., 10963 Berlin, Germany; matthias@kuske.de; 8 GAT-Grupo de Ativistas em Tratamentos, 1000-228 Lisboa, Portugal; ricardo.fuertes@gatportugal.org; 9 Checkpoint Athens, 105 54 Athens, Greece; schanos@athcheckpoint.gr; 10 AIDS Fondet, 1456 Copenhagen, Denmark; francoisdk@gmail.com

**Keywords:** early diagnosis, HIV, self-testing, men who have sex with men

## Abstract

We analyzed men who have sex with men (MSM) from Denmark, Germany, Greece, Portugal and Spain to identify who would choose HIV self-testing as their preferred testing method and assessed their preferred setting to acquire a self-testing kit and to confirm a reactive result. In 2016, we recruited an online sample of 3725 HIV-negative MSM. We used Poisson regression to identify factors associated with choosing self-testing as the preferred testing option. For those choosing it as their preferred option, we assessed the preferred settings to acquire a self-testing kit and to confirm a reactive result. Not being open about one’s sexual behaviors with men was associated with choosing self-testing as the preferred option, except in Greece; older age in Greece and Spain; reporting condomless anal intercourses (CAI) in Germany and Portugal; reporting one previous test in Greece; between 2 and 5 in Spain and with having been tested ≥ 12 months ago in Germany, Portugal and Spain. The internet (32.8%) was the preferred place to acquire a self-testing kit and primary care (34.0%) for confirmation purposes. Self-testing was highly valued, especially among individuals who were not open about their sexual behaviors with men. In certain countries, it was also associated with older age, CAI and being undertested.

## 1. Introduction

Men who have sex with men (MSM) are the group most at risk of acquiring HIV in European countries. In 2018, they represented 40% of all new diagnoses [1]. Among the main strategies to fight the epidemic is to promote testing to reduce time between infection and diagnosis [2,3]. From a patient perspective, early diagnosis and treatment improves health [4,5] and leads to a life expectancy similar to the general population [6]. The early initiation of treatment is also important from a public health perspective because highly active antiretroviral therapy (HAART) leads to a reduction in onward transmission by reducing viral load [7,8]. The promotion of earlier diagnosis is in fact a vital point of the 90-90-90 UNAIDS plan to end the epidemic: by 2030, 90% of all people living with HIV should be diagnosed, 90% of the diagnosed population should be on treatment and 90% of these should achieve viral suppression [2].

In light of the importance of early diagnosis, a number of initiatives have been rolled out to increase access to testing, both in clinical and non-clinical settings [9,10], but testing frequency among MSM [11] is far from meeting current recommendations of testing at least once every 12 months [12]. As a consequence, late diagnosis is still frequent in this group, and 41% of all the newly diagnosed individuals in Europe were diagnosed at a late stage of infection (CD4 count of <350 mm^3^) [1]. HIV self-testing (HIVST) is the most recent innovation introduced to increase testing frequency and reach those who remain undiagnosed. In this testing methodology, an individual takes a blood or oral sample, tests him/herself and obtains a result in under 20 min. Self-tests are not diagnostic and, if reactive, need to be confirmed. In Europe, HIVST started to be commercialized in the UK in 2014 and has spread to other countries, including Denmark [13] Germany [14] Portugal [15] and Spain [14].

This methodology offers confidential and anonymous testing in a convenient, quick and easy way, since it requires no appointments, no waits and no pre- and post-test discussion. HIVST could facilitate testing by lowering individual and structural barriers and by removing barriers related to the lack of anonymity and confidentiality [16,17]. On the other hand, the implementation of self-testing also poses a series of challenges. The sole use of self-testing could lead to missed opportunities of diagnosing other sexually transmitted infections (STI) [18] and, for those obtaining a reactive result, it is unclear if it would imply delays in the linkage to care process since there are limited data on this subject [17,19,20]. Additionally, HIVST needs to be considered in the context of Pre-exposure prophylaxis (PrEP). PrEP implies the use of an antiretroviral medication by people who are uninfected to prevent the acquisition of HIV. This preventive measure was approved by the European Medicines Agency in 2016 [21] and has been incorporated into national HIV prevention strategies since then [22]. According to the European Aids Clinical Society (EACS), it is recommended in HIV-negative MSM when condoms are not used consistently with casual partners or with HIV-positive partners who are not on treatment. A recent STI, use of post-exposure prophylaxis or chemsex may be markers of increased risk for HIV acquisition [23]. Patients on PrEP need to be tested for HIV every three months [23].

Previous studies among MSM have proven that although awareness of HIVST is modest [24,25], they report a high willingness to use this testing method [26,27,28]. However, all the aforementioned studies analyzed self-testing in isolation. There is a clear lack of studies that assess HIVST as a testing choice made in a context where testing is offered in several settings (distant, clinical and non-clinical) and using different tests (conventional and rapid; blood and oral based). We only found two UK-based studies [29,30] that approached preferences toward self-testing in this manner. In both, the support towards self-testing was lower than in those studies that assessed it independently.

For a successful incorporation of self-testing as a public health strategy, several implementation decisions need to be made to fully realize the potential of self-testing to increase testing rates. These decisions include the distribution of self-testing and establishing swift confirmation and linkage to care pathways for those obtaining a reactive test. In this sense, there is a lack of information on the preferences of MSM on where to obtain and confirm a self-test if reactive, which is needed so that informed choices can be made by decisions makers.

The aim of this study is to determine the proportion and characteristics of HIV-negative MSM recruited online in five European countries who would choose HIVST as their preferred testing choice over other already existing choices and assess which would be their preferred setting to obtain a self-testing kit and to confirm a reactive result.

## 2. Materials and Methods

This article was carried out in the context of the EURO HIV EDAT project (operational knowledge to improve HIV early diagnosis and treatment among vulnerable groups in Europe) (grant agreement number 2013 11 01). Further information can be found elsewhere [24]. The project was approved by the ethical committee of investigation and animal welfare of the Instituto de Salud Carlos III (CEI PI 52_2015-v2) and the Hospital Germans Trias i Pujol (PI-14-106).

### 2.1. Recruitment Procedures

We conducted an online cross-sectional study in 5 European countries (Denmark, Greece, Germany, Portugal and Spain) between April and December 2016. No sample size calculation was performed. We used non-probabilistic convenience sampling to recruit the largest possible sample of HIV-negative MSM. The dissemination of the survey was mainly performed through gay geo-spatial “dating” applications and websites that were previously identified by members of the research team who worked in Community Based Organizations (CBO). Geo-spatial applications are a widespread tool to meet new sexual partners and are an efficient way of recruitment [31]. However, we also advertised the study on websites of lesbian, gay, bisexual and transgender community organizations. Advertisement was performed through banners, direct messages and mailing lists.

Those who clicked on the promotional banner or link were directed to an introductory screen that included brief information on the aims of the project, its anonymity as well as funding and the partners involved. It also included a link to the EURO HIV EDAT project website for those interested in more information. Those who decided to participate gave their informed consent online by checking a box with the message *“I have read and understood the above information, in the country I live in I am old enough to legally have sex, and I want to participate”* before moving on to the first question. No incentives were offered for participation.

### 2.2. Data Collection Instrument

Data were collected through a self-administered online questionnaire. The questionnaire was designed in English and then translated into the different national languages by native speakers from the research team: Danish, German, Greek, Portuguese and Spanish. The questionnaire was also backtranslated using Google translate to check for mistakes and content discrepancies with the English version. The questionnaire included 90 items (generally showing 1 item per page), although not every participant had to answer all questions. The completion time was approximately 20 min. Respondents were able to review and change their answers using a “Back” button. Data collection was completely anonymous (no questions including personal identification were included) and confidential. Before launching the study, the instrument was piloted and revised by partners from all countries. No randomization of questionnaire items was performed.

We included questions to assess sociodemography, sexual risk behaviors, outness and history of previous STIs and testing experience. It also included a section to assess the preferences and patterns of use of the different testing options.

HIV-negative participants were asked about their pattern of use of the different testing options if all were available. The response options included were (1) “*I Would only use one testing option*”; (2) “*I would mainly use one, occasionally another one*”; (3) “*I would use one more frequently than the others, but would also use two others quite regularly*”; (4) “*Would use two options equally*”; (5) “*Would use three or more options*”. Following this question, we asked participants to choose which would be their preferred option from a list of response categories that included ten testing options: conventional testing at an HIV/STI specialized clinic/center; conventional testing at primary care; conventional testing at a private laboratory; rapid testing at a general health care service (primary care/emergency department); rapid testing at an HIV/STI specialized clinic/center; rapid testing at a pharmacy conducted by the pharmacist; rapid testing at a CBO or non-governmental organization; rapid testing at a bar/pub, club or sauna; home self-sampling and self-testing. For those who answered that they would use two testing options equally, they were also asked to note which those two methods would be.

For those who chose self-testing as their preferred testing option or selected it as one of the choices they would choose equally frequently (in the case of those who chose the “*would use two options equally option*”), we assessed which would be their preferred place to obtain a self-testing kit and to confirm a hypothetical reactive result. To assess the preferred place to obtain a kit, we used the following question “*Besides pharmacies, where would you like to be able to buy an HIV self-test?*”. Participants were asked to choose their preferred setting from the following list: (1) “*At supermarkets/drugstores*”, (2) “*At Community Based Organization or Non-Governmental Organization*”, (3) “*Through the internet*”, (4) “*Purchasing it by phone*”, (5) “*At vending machines*” and (6) “*Other [please specify]*”. We also included an option for those who felt that it should only be made available at pharmacies: (7) “*I think it should only be sold in pharmacies*”. For the assessment of the preferred setting for confirming a reactive self-test, we included the following question: “*If you obtained a reactive or indeterminate result with a self-test: where would you prefer to go for help and confirmation?*”. Participants were asked to choose their preferred setting from the following list: (1) “*Hospital or clinic*”, (2) “*HIV/STI testing service or clinic*”, (3) “*At the office of a medical specialist*”, (4) “*General practitioner/family doctor*”, (5) “*Private laboratory*”, (6) “*Pharmacy (where the test is performed by a pharmacist)*”, (7) “*At the emergency room of a hospital*”, (8) “*At a mobile unit*”, (9) “*Premises of a Community Based or Non-Governmental Organization*”, (10) “*In a bar/pub, club or sauna*” and (11) “*Elsewhere [please specify]*”.

### 2.3. Inclusion and Exclusion Criteria

We initially included HIV-negative MSM who resided in one of the participating countries (*N* = 3817), but we excluded 92 participants who had missing data on the set of questions that assessed preference towards testing options. Thus, our final sample comprised 3725 participants.

### 2.4. Data Analysis

We first describe the main characteristics of the sample by country of residence. The data included in the total column were weighed to adjust for the different country sample sizes. Weighting coefficients were calculated using data from EUROSTAT [32] and took into account the male population between 18 and 65 years old living in the participating countries in 2016.

To assess preference towards self-testing, we created a dichotomous variable. In the “yes” category, we included all those participants who chose it as their preferred option. In this category, we also included those who answered that they would use two testing options equally when one of the selected options was self-testing. In the “no” category, we included the rest of the participants.

We estimated the factors associated with choosing self-testing as the preferred option by conducting a Poisson regression mode with robust variance for each country. With the Poisson regression, we estimated crude and adjusted prevalence ratios (PRs), and 95% confidence intervals (95%CI). We chose Poisson regression with robust variance because it is a better alternative than logistic regression for the analysis of cross-sectional studies with binary outcomes [33,34]. We initially included all the relevant variables with a significance level ≤ 0.20 and used the minimum Akaike information criteria (AIC) and the minimum Bayesian Schwartz information criteria (BIC) to perform model comparisons and select the optimal one.

For those who chose self-testing as their preferred option, we assessed where they would prefer to obtain a self-testing kit aside from pharmacies, and which would be their preferred setting for confirming a reactive result, and presented the results by country.

## 3. Results

### 3.1. Main Characteristics of Participants

Forty-nine percent of our participants were 40 years of age or older (minimum percentage (min) in Greece (28.7%); maximum percentage (max) in Germany (56.0%)), and 90.6% were born in their country of residence. Approximately one-third (35.1%) lived in cities of under 100,000 inhabitants (min: 24.9% Greece; max: 43.9% Portugal), and 48.0% had not finished a university degree at the time of the survey (min: 32.2% Greece; max: 57.3% Germany) (Table 1).

Approximately 16.4% kept their sex lives with other men hidden or in total secrecy (min: 6.0% Denmark; max: 31.1% Greece) (Table 1).

Regarding sexual risk indicators, 62.7% (min: 48.6% Greece; max: 76.5% Denmark) reported having had condomless anal intercourse (CAI) in the last 12 months with at least one partner, and 42.4% (min: 37.0% Greece; max: 55.1% Denmark) had been diagnosed with an STI in the past (Table 1).

Overall, 17.5% (min: 13.3% Denmark; max: 19.6% Greece) had been tested only once in the past, and 37.4% (min: 26.4% Greece; max: 40.5% Germany) underwent testing more than 12 months ago (Table 1).

### 3.2. Proportion of Participants Who Chose Self-Testing as Their Preferred Testing Method and Associated Factors

Just over a third of our participants (34.4%, weighted percentage) chose self-testing as their preferred testing method. This proportion was highest among those answering from Germany (39.3%) and was followed by respondents from Portugal (35.0%), Denmark (29.4%), Spain (28.5%) and Greece (23.1%).

The results of the multivariable analysis for each of the participating countries can be found in Table 2. In Greece (PR: 5.5; 95%CI: 1.6–18.7) and Spain (PR: 1.5; 95%CI: 1.1–2.0), adjusted PRs were significantly higher among those aged ≥50 than in those aged <25. In Greece, the adjusted PR was also higher in those between 25 and 49 years of age (PR: 6.0; 95%CI: 2.0–17.7).

With the exception of Greece, adjusted PRs were higher among participants who kept their sex lives hidden or in total secrecy (Denmark (PR: 2.0; 95%CI: 1.2–3.4); Germany (PR: 1.4; 95%CI: 1.1–1.8); Portugal (PR: 1.6; 95%CI: 1.1–2.3); Spain (PR: 1.3; 95%CI: 1.1–1.6)). In Germany (PR: 1.5; 95%CI: 1.3–1.8) and Spain (PR: 1.2; 95%CI: 1.0–1.4), they were also significantly higher in those who reported living their lives “discreetly”.

In Germany (PR: 1.5; 95%CI: 1.2–1.8) and Portugal (PR: 1.5; 95%CI: 1.1–2.1), adjusted PRs were higher in participants who reported having CAI with ≥2 partners during the last 12 months compared to those who reported none. In Portugal, the adjusted PR also remained statistically significant in those reporting having had CAI with one partner (PR: 1.5; 95%CI: 1.0–2.1)

When compared to those who reported having undergone > 5 HIV tests in the past, adjusted PRs were significantly higher in participants from Greece with only one previous test (PR: 1.9; 95%CI: 1.2–3.2) and in participants answering from Spain with 2–5 previous tests (PR: 1.2; 95%CI: 1.0–1.4). In Germany (PR: 1.2; 95%CI: 1.0–1.5), Portugal (PR: 1.5; 95%CI: 1.1–1.9) and Spain (PR: 1.2; 95%CI: 1.1–1.4), those who tested >12 months ago had significantly higher adjusted PRs than those who had been tested <12 months ago.

### 3.3. Preferred Settings to Acquire a Self-Test and to Confirm a Reactive Result

When participants were asked about their preferred place to acquire a self-testing kit, the internet was chosen by 32.8% of all participants. It was the preferred option in Denmark (54.4%) and Germany (38.3%). Just over one-fourth of our sample (25.5%) thought that self-testing kits should only be sold in pharmacies. This was the most frequently chosen option in Greece (40.0%) and Spain (34.1%). Respondents from Portugal chose supermarkets/drugstores (27.8%) (Table 3).

Primary care was chosen as the preferred setting to confirm a reactive self-test by 34.0% of our participants and was the most common choice in Denmark (49.3%) and Germany (44.2%). Secondary health settings were the preferred option in Greece (36.9%) and Spain (37.7%), whereas respondents from Portugal chose sexual health clinics as their preferred setting (26.9%) (Table 3).

## 4. Discussion

When assessed in concurrence with already existing methodologies, self-testing was chosen as the preferred testing option by one-third of an online sample of MSM recruited in five European countries. Self-testing was especially valued in Germany and Portugal and less so in Spain and Greece. We found differences between countries, but it was especially popular among men who were not completely open about their sexual behaviors with men. Other subgroups in need of increased testing frequency, such as older age MSM, those who reported being undertested and at risk of acquiring HIV, also chose self-testing as their preferred option in some countries. Regarding preferences towards settings to acquire a self-test, participants chose the internet in Denmark and Germany and supermarkets/drugstores in Portugal. Respondents from Greece and Spain considered that self-testing should only be sold in pharmacies. Primary care (in Denmark and Germany), secondary health settings (Greece and Spain) and sexual health clinics (Portugal) were the preferred settings to confirm a reactive result.

Previous studies have reported the promising potential of HIVST to increase access to testing in MSM who report a high willingness to use this testing method [24,28,35,36,37]. However, these studies assessed HIVST in isolation with yes/no questions, such as “Would you have used self-testing in the past if already available?”. In a real-world scenario, the choice of HIVST is made against a wide variety of options, and the extent to which MSM would prefer to use HIVST over other testing methodologies has been rarely studied. The results of our study suggest that when preference towards self-testing is assessed along with the rest of the testing options, results are less significant than those reported in the aforementioned studies. Nevertheless, it was still a highly valued option, and just over a third of our participants would choose it over the rest of the testing options. Compared to our results, the only two studies we found that gauged testing preferences among MSM in the context of the already existing testing choices [29,30] reported lower proportions of participants choosing self-testing as their method of choice.

In our study, choosing self-testing over the rest of the options was especially frequent in MSM who were not completely open about their sexuality. This was observed in all countries except Greece. Those who are not open about their sexual behaviors with other men generally present lower testing rates than the rest of MSM [11]. Certain characteristics of remote methodologies, such as privacy, and not having to reveal sexual orientation or discuss sex-life with a healthcare professional, could be behind the popularity of self-testing in MSM who pertain to this subgroup.

Those involved in CAI favored self-testing above other testing options in Germany and Portugal but not in the rest of the countries. This could indicate that higher risk individuals could be comfortable with already existing options in certain countries but not in others. In this sense, a study conducted in the UK by Miners et al. [29] reported that the majority of MSM were comfortable with existing options and preferred them to self-testing. However, they also identified a much smaller group that contained individuals who were more likely to be at a high infection risk who preferred remote testing options. Recommendations in MSM involved in ongoing sexual risk behaviors is that they should test every three months [12]. Self-testing is convenient, easy and fast, and a population in need of frequent testing could favor it precisely due to these reasons. However, if it were to be adopted by high-risk individuals, there should be effective alternatives to ensure the testing frequency of other STIs which, in recent years, have increased among European MSM [38,39,40].

In Germany, Portugal and Spain, self-testing was favored above other options by undertested individuals. Additionally, in Greece (and in Spain), it was also favored by those reporting less lifetime testing. Both results suggest that self-testing could lead to increased testing among undertested populations. Although the efficacy of self-testing in increasing testing rates has been demonstrated in randomized control trials [18,41], its effectiveness in a real-life scenario still needs to be assessed.

We found no studies that assessed preferences toward settings to acquire self-testing kits and to confirm a reactive result. This evidence is important because it provides insightful information to those in charge of designing the implementation of HIVST strategies on how MSM would prefer to engage with this technology. In our study, the internet was chosen as the preferred option to acquire a self-test. The internet has become an accepted way to purchase products and services, and our hypothesis is that this option would be even more frequent today, especially if we consider the buying methods that COVID-19 lockdowns have imposed in all societies. Buying pharmacy products online is probably no exception. Being able to acquire self-testing kits online increases accessibility and, when incorporating HIVST in national testing policies, countries need to first consider the internet. However, unmarked/unapproved testing kits are a safety risk that needs to be taken into account, since they could perform suboptimally and could result in false positive and false negative results.

Timely linkage to care following HIV diagnosis is also critical, since late access can result in worst patient outcomes [42]. In particular, with remote testing methodologies, countries need to create robust confirmation routes to ensure optimal linkage to care. In this sense, knowing participants’ preferences to seek confirmation of testing could help establish swift linkage to care pathways. In our sample, we found that primary care settings (Denmark and Germany) and secondary health settings (Spain and Greece) were the preferred options. The only exception was found in respondents from Portugal who chose sexual health clinics, although NGO/CBOs and secondary health settings followed closely.

Our results are not without limitations. Our study included participants that have not experienced HIVST themselves and, therefore, are based in a hypothetical scenario. In 2016, self-testing was yet to be approved in all participating countries, and the attitude towards this testing option could have changed in those who have now used it. Participants included in the study were recruited mainly through gay dating websites. Although using websites/apps of this nature is common among MSM [43,44], the generalization of our results to other MSM should be made with caution. Additionally, the introduction of PrEP has re-shaped the HIV prevention scenario. PrEP was not assessed in the present study since, at the time of data collection, it was still not approved in the European Union. PrEP for MSM is only recommended in individuals at high risk of acquiring HIV. MSM who are on PReP should be tested every three months using fourth generation laboratory testing [23,45]. The World Health Organization (WHO) states that testing kits for HIV self-testing should not replace facility-based testing during PrEP use [46]. In this sense, using HIVST is not the ideal option for this population, but it could, nevertheless, help meet recommendations in those who have trouble accessing facility-based services for PrEP follow up and testing [47,48] and in certain scenarios, such as COVID-19 lockdowns [49].

## 5. Conclusions

When assessed jointly with the other existing testing options, HIVST still remains a popular option among MSM recruited online in five European countries. This testing option was especially favored by those who are not completely open about their sexuality. In certain countries, subgroups such as high-risk and undertested MSM chose self-testing as their preferred option. This underlines the popularity of a highly accessible and convenient way of testing which could facilitate testing in the aforementioned subgroups but also represents future challenges, such as ensuring optimal confirmation and linkage to care that need to be monitored to further assess the capacity of self-testing to promote early access to treatment. Additionally, further studies need to be performed to understand how HIVST is incorporated in the testing habits of PrEP users.

## Figures and Tables

**Table 1:** Sociodemographic profile, outness, sexual behaviors, history of sexually transmitted infections (STI) and testing history.

	Denmark (*N* = 248)		Germany (*N* = 794)		Greece (*N* = 363)		Portugal (*N* = 417)		Spain (*N* = 1903)		Total (Weighted) (*N* = 3725)
	*N*	%	*N*	%	*N*	%	*N*	%	*N*	%	%
**Age**											
<25	26	10.5	43	5.4	61	16.8	34	8.2	198	10.4	8.0
25–29	31	12.5	85	10.7	61	16.8	53	12.7	314	16.5	13.0
30–34	30	12.1	95	12.0	59	16.3	65	15.6	301	15.8	13.6
35–39	39	15.7	126	15.9	78	21.5	80	19.2	291	15.3	16.3
40–49	78	31.5	227	28.6	74	20.4	107	25.7	505	26.5	27.3
≥50	44	17.7	218	27.5	30	8.3	78	18.7	294	15.4	21.7
**Place of birth**											
In country of current residence	212	85.8	732	92.4	339	97.1	360	86.7	1644	87.2	90.6
Europe	22	8.9	40	5.1	4	1.1	14	3.4	78	4.1	4.5
Others	13	5.3	20	2.5	6	1.7	41	9.9	163	8.6	4.9
**Number of inhabitants in place of residence**											
≥1,000,000	88	35.6	198	25.0	201	55.7	88	21.2	666	35.1	30.2
100,000–999,999	84	34.0	286	36.1	70	19.4	145	34.9	675	35.5	34.7
10,000–99,999	45	18.2	213	26.9	71	19.7	126	30.4	406	21.4	24.7
<10,000	30	12.1	95	12.0	19	5.3	56	13.5	153	8.1	10.5
**Education**											
Up to upper secondary education	100	40.5	264	33.3	46	12.7	113	27.2	479	25.2	29.4
Post-secondary non-tertiary education	10	4.0	190	24.0	71	19.6	27	6.5	251	13.2	18.6
University education	137	55.5	339	42.7	246	67.8	276	66.3	1170	61.6	52.0
**Lives sex life with men…**											
Openly	190	76.6	428	54.0	79	21.8	85	20.4	907	47.7	48.6
Discreetly	43	17.3	236	29.8	171	47.1	227	54.4	754	39.6	35.0
Hidden/In total secrecy	15	6.0	129	16.3	113	31.1	105	25.2	241	12.7	16.4
**Number of partners with CAI (last 12 months)**											
None	57	23.5	288	37.1	183	51.4	134	33.1	688	37.0	37.3
1	63	25.9	222	28.6	116	32.6	126	31.1	612	32.9	30.2
2–4	66	27.2	170	21.9	43	12.1	101	24.9	372	20.0	21.1
≥5	57	23.5	96	12.4	14	3.9	44	10.9	189	10.2	11.4
**History of STI**											
STI diagnosis in the last 12 months	34	14.0	72	9.4	41	11.7	59	14.9	203	11.0	10.5
STI diagnosis > 12 months ago	100	41.2	244	31.8	88	25.2	112	28.2	609	33.0	31.9
No STI diagnosis	109	44.9	451	58.8	220	63.0	226	56.9	1031	55.9	57.6
**Number of HIV test (ever)**											
1	33	13.3	133	16.8	71	19.6	73	17.5	358	18.8	17.5
2–5	93	37.5	382	48.1	160	44.1	189	45.3	894	47.0	46.9
6–9	43	17.3	127	16.0	54	14.9	67	16.1	317	16.7	16.2
≥10	79	31.9	152	19.1	78	21.5	88	21.1	334	17.6	19.4
**Time since last HIV test**											
≤3 months ago	68	27.4	172	21.7	147	40.5	92	22.1	488	25.7	24.4
3–12 months ago	97	39.1	299	37.8	120	33.1	189	45.4	733	38.6	38.2
1–2 years ago	46	18.5	135	17.0	52	14.3	60	14.4	338	17.8	17.0
> 2 years ago	37	14.9	186	23.5	44	12.1	75	18.0	339	17.9	20.4

**Table 2:** Proportion of participants who chose self-testing as their preferred testing option (if all available) and associated factors.

	Denmark (*N* = 248)		Germany (*N* = 794)		Greece (*N* = 363)		Portugal (*N* = 417)		Spain (*N* = 1903)	
	P (%)	PR (95%CI)	P (%)	PR (95%CI)	P (%)	PR (95%CI)	P (%)	PR (95%CI)	P (%)	PR (95%CI)
**Overall**	29.4		39.3		23.1		35.0		28.5	
**Age**										
≥50	25.0	1.0 (0.4–2.4)	39.4	1.1 (0.7–1.7)	26.7	5.5 (1.6–18.7)	26.9	0.8 (0.4–1.4)	33.0	1.5 (1.1–2.0)
25–49	31.5	1.3 (0.7–2.8)	39.6	1.1 (0.8–1.8)	26.8	6.0 (2.0–17.7)	37.4	1.2 (0.7–1.9)	28.6	1.3 (1.0–1.7)
<25	23.1	1.0	34.9	1.0	4.9	1.0	32.4	1.0	21.7	1.0
**Lives sex life with men…**										
Hidden/In total secrecy	53.3	2.0 (1.2–3.4)	45.7	1.4 (1.1–1.8)	29.2		47.6	1.6 (1.1–2.3)	35.7	1.3 (1.1–1.6)
Discreetly	32.6	1.2 (0.7–2.0)	48.3	1.5 (1.3–1.8)	21.1		30.8	1.0 (0.7–1.5)	31.3	1.2 (1.0–1.4)
Openly	26.8	1.0	32.2	1.0	19.0		30.6	1.0	24.4	1.0
**Number of partners with CAI (last 12 months)**										
≥2	33.3		48.1	1.5 (1.2–1.8)	14.0		40.0	1.5 (1.1–2.1)	30.3	
1	28.6		35.1	1.1 (0.9–1.4)	24.1		38.1	1.5 (1.0–2.1)	27.1	
None	24.6		34.4	1.0	24.0		28.4	1.0	28.3	
**Number of HIV test (ever)**										
1	39.4		43.6		31.0	1.9 (1.2–3.2)	42.5		31.6	1.2 (0.9–1.5)
2–5	23.7		40.3		23.8	1.4 (0.9–2.2)	36.5		30.6	1.2 (1.0–1.4)
>5	31.1		35.8		18.2	1.0	29.7		24.0	1.0
**Time since last HIV test**										
>12 months ago	32.5		44.5	1.2 (1.0–1.5)	31.3		45.9	1.5 (1.1–1.9)	34.6	1.2 (1.1–1.4)
≤12 months ago	27.9		35.7	1.0	20.2		29.9	1.0	25.1	1.0

P: prevalence. PR: adjusted prevalence ratio. 95%CI: 95% confidence interval.

**Table 3:** Preferred setting to acquire an HIV self-testing kit and to confirm a reactive result in men who have sex with men who chose self-testing as their preferred testing option.

	Denmark (*N* = 73)		Germany (*N* = 312)		Greece (*N* = 84)		Portugal (*N* = 146)		Spain (*N* = 543)		Total (Weighted) (*N* = 1158)
	*N*	%	*N*	%	*N*	%	*N*	%	*N*	%	%
**In addition to pharmacies: where would you like to be able to acquire the HIV self-test?**											
Through the internet	31	54.4	100	38.3	12	17.1	24	26.7	101	25.1	32.8
It should only be sold in pharmacies	14	24.6	52	19.9	28	40.0	16	17.8	137	34.1	25.5
Supermarkets/drugstores	4	7.0	63	24.1	14	20.0	25	27.8	85	21.1	22.6
Vending machines	2	3.5	16	6.1	10	14.3	17	18.9	58	14.4	9.9
NGO/CBO	2	3.5	24	9.2	0	0.0	3	3.3	17	4.2	6.5
Others	4	7.0	6	2.3	6	8.6	5	5.6	4	1.0	2.7
**Preferred setting to confirm** **a reactive self-test**											
Primary care	36	49.3	137	44.2	2	2.4	24	16.6	133	24.6	34.0
Secondary health setting	11	15.1	94	30.3	31	36.9	35	24.1	204	37.7	32.0
Sexual health clinic	18	24.7	36	11.6	30	35.7	39	26.9	154	28.5	19.7
In a NGO/CBO (office or mobile unit)	8	11.0	30	9.7	8	9.5	38	26.2	36	6.7	9.8
Private laboratory	0	0.0	7	2.3	10	11.9	7	4.8	10	1.8	2.9
Others	0	0.0	6	1.9	3	3.6	2	1.4	4	0.7	1.6

NGO: non-governmental organization. CBO: community-based organization.

## Data Availability

The data presented in this study are available on reasonable request from the corresponding author.
